# Transcription Factor Elf3 Modulates Vasopressin-Induced Aquaporin-2 Gene Expression in Kidney Collecting Duct Cells

**DOI:** 10.3389/fphys.2019.01308

**Published:** 2019-10-18

**Authors:** Shu-Ting Lin, Chia-Ching Ma, Kuang-Ting Kuo, Yin-Fang Su, Wei-Ling Wang, Tzu-Hsien Chan, Shih-Han Su, Shih-Che Weng, Chian-Huei Yang, Shuei-Liong Lin, Ming-Jiun Yu

**Affiliations:** ^1^Institute of Biochemistry and Molecular Biology, College of Medicine, National Taiwan University, Taipei, Taiwan; ^2^Department of Parasitology, College of Medicine, National Taiwan University, Taipei, Taiwan; ^3^Graduate Institute of Physiology, College of Medicine, National Taiwan University, Taipei, Taiwan

**Keywords:** Elf3, Ets, vasopressin, aquaporin-2, transcription, collecting duct

## Abstract

Aquaporin-2 (AQP2) is a molecular water channel protein responsible for water reabsorption by the kidney collecting ducts. Many water balance disorders are associated with defects in AQP2 gene expression regulated by the peptide hormone vasopressin. Here, we studied roles of Elf3 (E26 transformation-specific (Ets)-related transcription factor 3) in AQP2 gene expression in the collecting duct cells (mpkCCD). Vasopressin increased AQP2 mRNA and protein levels without affecting AQP2 mRNA degradation, indicative of transcriptional regulation. Elf3 knockdown and overexpression, respectively, reduced and increased AQP2 gene expression under basal and vasopressin-stimulated conditions. However, the vasopressin-to-basal ratios of AQP2 gene expression levels remained constant, indicating that Elf3 does not directly mediate vasopressin response but modulates the level of AQP2 gene expression inducible by vasopressin. The Elf3-modulated AQP2 gene expression was associated with AQP2 promoter activity, in line with Elf3’s ability to bind an Ets element in the AQP2 promoter. Mutation in the Ets element reduced both basal and vasopressin-stimulated AQP2 promoter activity, again without affecting vasopressin-to-basal ratios of the AQP2 promoter activity. Lithium chloride reduced both Elf3 and AQP2 mRNA in the mpkCCD cells as well as in mouse kidney inner medulla. We conclude that Elf3 modulates AQP2 promoter activity thereby gauging vasopressin-inducible AQP2 gene expression levels. Our data provide a potential explanation to lithium-induced nephrogenic diabetes insipidus where lithium reduces Elf3 and hence AQP2 abundance.

## Introduction

Vasopressin is a peptide hormone released from the pituitary gland upon elevated serum osmolality (Knepper et al., 2015). It acts on the kidney collecting ducts to increase water permeability and hence osmotic water reabsorption. Vasopressin does so via two mechanisms. In the short-term responses, vasopressin triggers shuttling of the molecular water channel protein aquaporin-2 (AQP2) to and from the apical/luminal plasma membrane of the collecting duct cells thereby increasing water permeability (Wade et al., 1981; Nielsen et al., 1995). In the long-term responses, vasopressin increases overall AQP2 gene expression at the mRNA and protein levels (DiGiovanni et al., 1994; Hayashi et al., 1994; Saito et al., 1999). Many water balance disorders are associated with dysregulation in the long-term vasopressin responses for example hypokalemia (Marples et al., 1996), hypercalcemia (Bustamante et al., 2008), and lithium-induced nephrogenic diabetes insipidus (Bockenhauer and Bichet, 2015; Knepper et al., 2015; Alsady et al., 2016). Understanding the long-term vasopressin-regulated AQP2 gene expression is fundamental to the physiology and pathophysiology of water balance.

In principle, AQP2 protein abundance in the collecting duct cells can be altered by vasopressin via processes that increase or decrease AQP2 protein abundance (Radin et al., 2012). For example, vasopressin can alter AQP2 protein abundance by altering its removal from the collecting duct cells via exosome excretion (Kanno et al., 1995; Pisitkun et al., 2004) or degradation by lysosome or proteasome (Kamsteeg et al., 2006; Sandoval et al., 2013). Vasopressin can also alter AQP2 protein abundance by altering the abundance and availability of AQP2 mRNA for translation (Hasler et al., 2006; Khositseth et al., 2011). Among the processes that could alter AQP2 mRNA levels, most researchers favor mRNA transcription that increases AQP2 mRNA (Hozawa et al., 1996; Matsumura et al., 1997; Uchida et al., 1997; Yasui et al., 1997).

The proximal AQP2 promoter region contains a number of *cis*-regulatory transcription factor binding elements (Yu et al., 2009). Early studies have shown that the cyclic AMP response element and the AP-1 element respond to vasopressin or cyclic AMP in cultured cells (Hozawa et al., 1996; Matsumura et al., 1997; Yasui et al., 1997). Knockout of protein kinase A catalytic subunits in the collecting cell model (mpkCCD) diminishes AQP2 gene expression inducible by vasopressin, reinforcing the importance of cylic AMP in AQP2 gene expression (Isobe et al., 2017). The GATA binding element was shown to positively or negatively regulate AQP2 promoter activity also in cultured cells (Furuno et al., 1996; Rai et al., 1997; Uchida et al., 1997). Recently, Yu et al. using a mouse model demonstrated unequivocally a role of the GATA2 transcription factor in AQP2 transcription (Yu et al., 2014). Thus, there is a continuing interest in identifying the transcription factors responsible for AQP2 gene expression.

Based on a number of modern quantitative systems studies (Yu et al., 2009; Tchapyjnikov et al., 2010; Schenk et al., 2012), we hypothesized a potential role of the transcription factor Elf3 in vasopressin-induced AQP2 gene expression. Recently, Grassmeyer et al. (2017) showed that Elf5 deficiency resulted in a small but significant reduction in AQP2 gene expression in a conditional knockout mouse model. This small significant reduction could be due to compensatory effects of other transcription factor such as Elf3 in the same Ets IIa family as Elf5 (Wei et al., 2010). Following our systems discovery, here we used the methods of molecular biology and biochemistry to show that Elf3 knockdown profoundly reduced AQP2 gene expression. To our surprise, Elf3 knockdown reduced both basal and vasopressin-induced AQP2 gene expression levels, without affecting the basal-to-vasopressin AQP2 expression ratio. Similar results were found under Elf3 overexpression conditions. Thus, Elf3 modulates the responsiveness of the AQP2 promoter to vasopressin and thereby gauges AQP2 gene expression levels inducible by vasopressin. In addition, our results also provide a potential mechanism to lithium-induced nephrogenic diabetes insipidus in a mouse model.

## Materials and Methods

### Cell Culture

**FIGURE 1:**
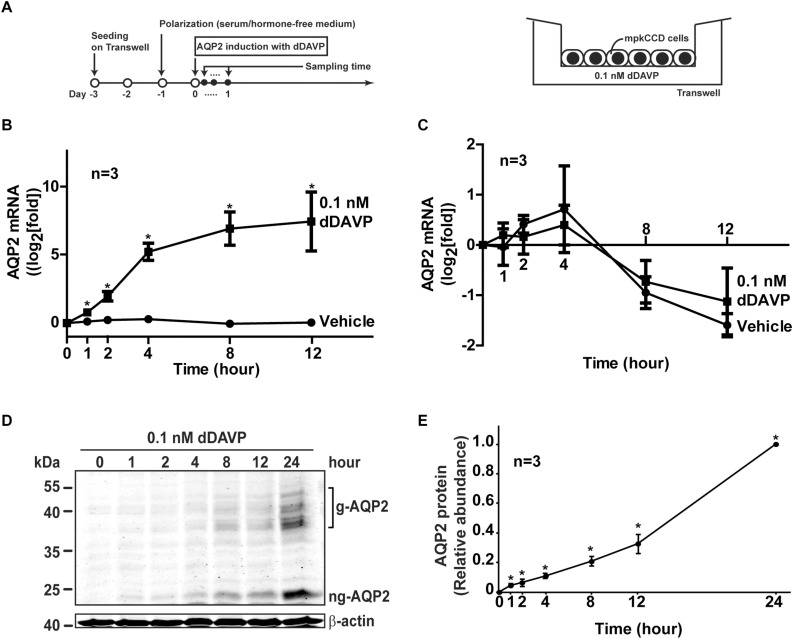
Vasopressin increased AQP2 gene expression in the mpkCCD cells. **(A)** The experimental protocol for cell polarization and endogenous AQP2 induction. **(B)** Time-course quantitative real time PCR measurements of AQP2 mRNA in the mpkCCD cells exposed to vehicle or vasopressin analog (0.1 nM dDAVP). Values are means ± SE, adjusted for loading with the RPLP0 mRNA levels and normalized against the values at time zero. Asterisk indicates significance (*p* < 0.05, *t*-test) against the values of the vehicle-treated cells at the same time point. **(C)** Time-course measurements of AQP2 mRNA in the mpkCCD cells in the presence of actinomycin D. Polarized mpkCCD cells were induced to express AQP2 mRNA (24 h). The cells were then treated with actinomycin D (2 μM) in the absence or presence of dDAVP for the indicated time before the AQP2 mRNA measurements. **(D,E)** Representative and summary of immunoblotting for AQP2 protein in the mpkCCD cells in response to dDAVP. Values are means ± SE, adjusted for loading with β-actin levels and normalized against the values at 24 h. Asterisk indicates significance (*p* < 0.05, *t*-test) against the values at 0 h. Two-way ANOVA was used to assess time-dependent effects of dDAVP vs. vehicle treatment. Respectively, g-AQP2 and ng-AQP2 indicate glycosylated and non-glycosylated AQP2.

The mpkCCD cells used in the present study originated in Alain Vandewalle’s laboratory (Duong Van Huyen et al., 1998). We recloned their cells for the highest AQP2 expression level (Yu et al., 2009). The recloned cells were cultured in a high glucose (17.5 mM) DMEM/F12 medium (Cat. 12400, Thermo Fisher Scientific) supplemented with dexamethasone (50 nM), epidermal growth factor (1.7 nM), insulin (870 nM), sodium selenite (60 nM), transferrin (64 nM), tri-iodotyrosine (1 nM), and 2% serum as described previously (Loo et al., 2013). Figure 1A shows our protocol to induce endogenous AQP2 gene expression with the vasopressin V2 receptor-specific analog dDAVP (1-deamino-8-D-arginine vasopressin). Cells (1 × 10^5^ per cm^2^, between 18 and 32 passages) were seeded on membrane supports (Transwell, 0.4 μM pore size, Corning Costar) and grown with symmetrical mediums in the apical and basal sides for 3 days. One day prior to the experiments, the cells were deprived of serum and supplements in both mediums to facilitate cell polarization (i.e., transepithelial electric resistance >5000 Ω.cm^2^). Thereafter, 0.1 nM (100 pM) dDAVP was added to the basal medium to induce AQP2 gene expression. This dDAVP concentration is found under maxiaml antidiuresis conditions i.e., 20–30% isoosmotic volume depletion (Bankir, 2001). In the mpkCCD cells, it produces a satisfactory amount of AQP2 easily detectable with immunoblotting (Hasler et al., 2002; Yu et al., 2009). HEK293T cells used for packaging small hairpin RNA (shRNA)-carrying lentivirus were maintained in the DMEM medium containing 10% fetal bovine serum.

### Quantitative Real-Time Polymerase Chain Reaction

Total RNA was extracted with the TRIzol reagent (Invitrogen). After removal of genomic DNA via DNase I digestion, first strand cDNA was produced via reverse transcription using an oligo-dT primer. Quantitative polymerase chain reaction (qPCR) was carried out with the KAPA SYBR FAST qPCR Master Mix (Kapa Biosystems) using gene-specific primers (Table 1) and the StepOnePlus^TM^ Real-Time PCR system (Thermo Fisher Scientific). The PCR conditions included an initial denature at 95°C for 10 min followed by 40 cycles of denature at 95°C for 15 s and annealing/extension at 60°C for 60 s. All samples were analyzed in triplicates. The ribosomal protein large P0 (RPLP0) mRNA levels were measured as internal controls. Reactions without template were included as negative controls.

**TABLE 1:** Oligonucleotide primers used in this study.

**Primer**	**Sequence**
**AQP2 promoter (ChIP-PCR)**	
Forward 1	GACCTTTTGCCTTGGAAATT
Reverse 1	CCTTCTCTTTGACTAAAAGGC
Forward 2	GTTTTCCATCCTCTTCCAG
Reverse 2	CAATGCCTTACCCCAAAC
Forward 3	ACTGCAAGTCACAACACAAC
Reverse 3	CCATTGTGAGACTTGACTGC
**AQP2 promoter cloning**	
Forward	CACCGGTACCCACCAAGACTAGAGGTTACT
Reverse	CACCAAGCTTCGGAGAGGCTAGACTGTGGG
**AQP2 quantification**	
Forward	CCTCCTTGGGATCTATTTCA
Reverse	CAAACTTGCCAGTGACAACT
**Elf3 expression vector cloning**	
Forward	CACCATGGCTGCCACCTGTGAGATCA
Reverse	TTCCGACTCTCTCCAACCTCTTCTT
**Elf3 isoform 1 quantification**	
Forward	GCCATGTACAGCTCAGAAGA
Reverse	AGGATCAGTCCTGTCCCTTT
**Elf3 isoform 2 quantification**	
Forward	GCCATGTACAGCTCAGAAGA
Reverse	GCTTGCCTTCTCTGGACCTT
**Elf3 quantification**	
Forward	TGATGTCTCCACTGCAAGGA
Reverse	TAGTCAGGAAAGCCATCTCT
**ETS mutation**	
Forward	GACCATCCAGTCCGATTCAAGGCAGCGCCCACA
Reverse	TGTGGGCGCTGCCTTGAATCGGACTGGATGGTC
**RPLP0 (ribosomal protein large P0) quantification**	
Forward	AGATCGGGTACCCAACTGTT
Reverse	GGCCTTGACCTTTTCAGTAA

### Immunoblotting

Immunoblotting was described previously (Loo et al., 2013). The primary antibodies were: AQP2 [sc-9882, Santa Cruz and (Hoffert et al., 2008)], β-actin (A5441, Sigma), GAPDH (glyceraldehyde 3-phosphate dehydrogenase, GTX627408, GeneTex), histone H2A (GTX628789, GeneTex), and V5-tag (GTX117997, GeneTex). The secondary antibodies were IRDye 680 or 800-conjugated (Li-Cor). Proteins were visualized and quantified with the Li-Cor Odyssey scanner and software. Protein abundance was normalized with β-actin staining.

### Small Hairpin RNA-Mediated Gene Knockdown

Plasmids containing Elf3-specific shRNA sequence (TRCN0000350801 GGTCGGATCATCCCTAATTTA targeting the 3′-UTR of Elf3 isoform 1 and 2) or non-targeting sequences (TRCN0000231693 CTTCGAAATGTCCGTTCGGTT or TRCN0000208001 CCGGACACTCGAGCACTTTTTG) were obtained from the RNAi core facility in the Academia Sinica, Taiwan. To produce lentivirus carrying the shRNA sequence, the HEK293T cells were seeded at 60% confluence in a 6-cm dish 1 day prior to transfection with the shRNA plasmid (4 μg), the packaging plasmid pCMV-ΔR8.91 (containing gag, pol and rev genes, 3.6 μg), and the envelope plasmid pMD.G (expressing VSV-G protein, 0.4 μg) dissolved in 250 μl serum-free medium plus 12 μl transfection reagent (T-Pro NTR II, T-Pro Biotechnology, New Taipei City, Taiwan). After 48 h of transfection, shRNA-containing lentivirus was collected in the supernatant of the culture medium after spun at 1,100 × g for 5 min. For shRNA-mediated gene knockdown, 3 × 10^5^ mpkCCD cells were seeded in a 6-cm dish 1 day prior to infection with the shRNA-containing lentivirus (1 ml lentivirus-containing medium plus 2 ml fresh medium and 24 μl 1 mg/ml polybrene). Stable gene knockdown cells were selected with puromycin (2.5 μg/ml).

### V5-Tagged Elf3 Expression Vector Construction

Elf3 isoform 1 and 2 cDNA fragments were PCR amplified from the cDNA library of the mpkCCD cells using specific primers (Table 1). The PCR products were cloned into the pENTR^TM^/SD/D-TOPO entry vector before shuttled to the pcDNA-DEST40 expression vector using the Invitrogen Gateway cloning system to generate V5-tagged Elf3 isoform 1 and 2 expression vectors.

### Confocal Immunofluorescence Microscopy

Confocal immunofluorescence microscopy was carried out as described previously (Loo et al., 2013). Confocal images were acquired with a Leica TCS SP5 microscope and processed with the Leica LAS-AF software (Leica Microsystems). The AQP2 and V5 antibodies were described above. The secondary antibodies were Alexa 488 fluorophore-conjugated (Invitrogen). Nuclei were stained with DAPI (4′,6-diamidino-2-phenylindole).

### Fractionation of Nuclear and Cytosolic Fraction

Cells were solubilized in 0.4 ml NC buffer (10 mM HEPES, 10 mM KCl, and 0.2 mM EDTA) containing protease inhibitor (539134, Calbiochem) and phosphatase inhibitor (524625, Calbiochem). The cell lysate was put on ice for 15 min before NP40 (to a final concentration of 0.6%) was added. The mixture was vortexed for 20 s and spun at 1,500 xg for 5 min at 4°C before the supernatant (the cytosolic fraction) was collected. The pellet was washed with 1 mL NC buffer and centrifuged at 1,500 xg for 5 min in 4°C. The pellet was re-suspended in 0.1 ml NC buffer and sonicated. After centrifugation at 16,000 xg for 10 min at 4°C, the supernatant (the nuclear fraction) was collected for analysis.

### AQP2 Promoter Activity Assay

A 1,000 base-pair AQP2 promoter DNA sequence containing the Ets element (Yu et al., 2009) was PCR amplified using specific primers (Table 1) and inserted into the pGL3 firefly luciferase vector (E1751, Promega) (Kuo et al., 2018). The AQP2 promoter-reporter vector was transfected into the mpkCCD cells. After experimental treatments, cell lysates were collected for luciferase activity assay. The Renilla luciferase reporter vector pRL-TK (E2241, Promega) was co-transfected for normalization purposes.

### Electrophoretic Mobility Shift Assay

Detailed procedures were described previously (Wang et al., 2007). Double-stranded AQP2 promoter oligonucleotide (−444 to −416 upstream to the transcription start site) containing the Ets element (Yu et al., 2009) was synthesized and labeled with γ-^32^P radioactive isotope. Nuclear extract of V5-tagged Elf3-expressing mpkCCD cells was incubated with the AQP2 promoter oligonucleotide before the mixture was subjected to acrylamide gel electrophoresis and autoradiography. Competitive binding was performed by including a non-labeled AQP2 promoter oligonucleotide at 200 folds in excess of the radioisotope-labeled oligonucleotide. Alternatively, a horseradish peroxidase-conjugated V5 antibody (Bethyl Laboratories, United States) was included to interfere the binding between the V5-tagged Elf3 and the labeled AQP2 promoter oligonucleotide (Leers-Sucheta et al., 1997). A non-specific IgG was used as a negative control.

### Chromatin Immunoprecipitation Coupled With Polymerase Chain Reaction

Chromatin immunoprecipitation (ChIP) was performed with a SimpleChIP Enzymatic Chromatin IP kit (Cat. 9003, Cell Signaling Technology). Briefly, V5-tagged Elf3 isoform 1-expressing mpkCCD cells were cross-linked with 1% formaldehyde at room temperature for 10 min. After formaldehyde was quenched with 125 mM glycine, chromatin was digested with micrococcal nuclease for 20 min. The digested chromatin was then briefly sonicated to lyse nuclear membranes. The lysate was then incubated with an Elf3 antibody (A13489, ABclonal), V5 antibody (GTX117997, GeneTex), or control IgG antibody at 4°C overnight. The mixture was incubated with ChIP-grade protein G magnetic beads at 4°C for 2 h. The chromatin DNA was then eluted and purified for polymerase chain reaction with primer sets specific to the AQP2 promoter region.

### Animal Study

Animal experiments were conducted under the auspices of the animal protocol 20130236 approved by the Laboratory Animal Center in the College of Medicine, National Taiwan University. Pathogen-free male C57BL/6 mice were kept in metabolic cages and provided with *ad libitum* water and mouse chow without or with LiCl (1.7 g/kg) for 14 days (Gomez-Sintes and Lucas, 2010). One day prior to sacrifice, mouse blood pressure was measured by the tail using a non-invasive system (Visitech Systems Inc.). Urine was collected during the 24 h. Mice were then weighed and anesthetized. Blood samples were collected through the inferior vena cava. The mice were sacrified and the kidneys were removed. The kidney inner medulla were excised and analyzed.

### Statistics

To assess effects of treatment vs. control, the Student’s *t*-test was used to determine statistical significance. To assess effects of treatment vs. control in time-course and/or dose-dependent experiments, one-way or two-way analyses of variance (ANOVA) was used. To assess multiple differences between two groups, the Tukey’s test was used after examining equality of group variances with the Brown-Forsythe test. In general, we considered a significant difference when the *p* value is less than 0.05.

## Results

### Vasopressin Increased AQP2 Gene Expression in the mpkCCD Cells

Figure 1A illustrates our Transwell setup and experimental protocol used to induce endogenous AQP2 gene expression in the mpkCCD cells with dDAVP. Figure 1B shows the time-course AQP2 mRNA expression profile in the mpkCCD cells in response to dDAVP. As short as 1 h after dDAVP stimulation, the AQP2 mRNA level increased significantly to about 1.8 folds and plateaued at about 8.0 folds at 8 h. In contrast, the AQP2 mRNA levels remained low at all time points under the vehicle control conditions. Two-way ANOVA showed no interaction between dDAVP and vehicle treatment and time-dependent increases in AQP2 mRNA abundance upon dDAVP treatment. No significant time-dependent changes were found under the vehicle conditions. The dDAVP-induced AQP2 mRNA increases could be attributed to enhanced AQP2 mRNA transcription because the rates of AQP2 mRNA decrease were not significantly different regardless dDAVP when mRNA synthesis was inhibited by actinomycin D (Figure 1C). The dDAVP-induced AQP2 mRNA increases were paralleled with significant increases in the AQP2 protein level in a time-dependent manner (Figures 1D,E).

### Elf3 Knockdown Reduced Basal and Vasopressin-Induced AQP2 Gene Expression

**FIGURE 2:**
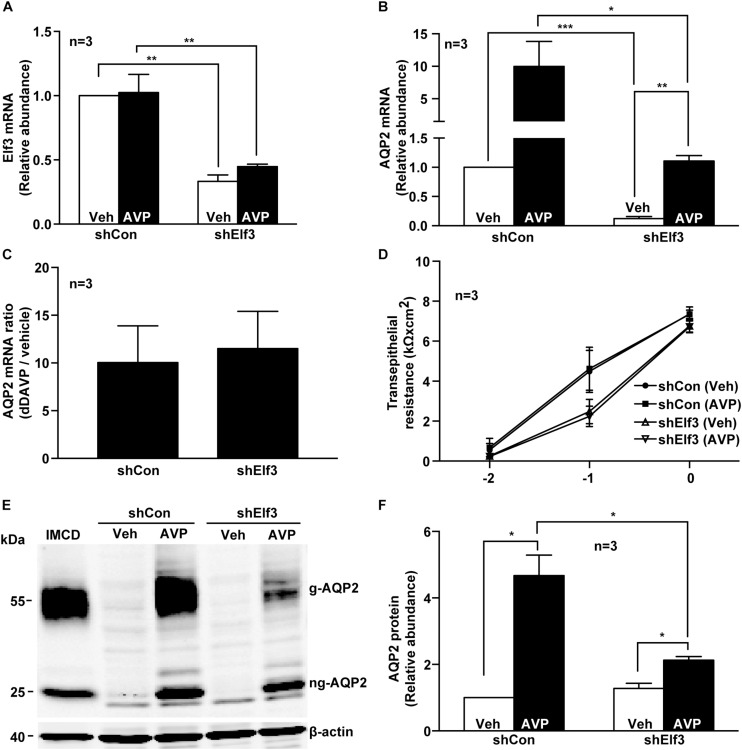
Elf3 knockdown reduced basal and vasopressin-induced AQP2 gene expression in the mpkCCD cells. **(A)** Elf3 and **(B)** AQP2 mRNA levels in the control (shCon) or stable Elf3 knockdown cells in response to vehicle (Veh) or 0.1 nM dDAVP (AVP) stimulation for 8 h. Values are means ± SE, adjusted for loading (RPLP0 mRNA levels) and normalized against the values of the control cells under the vehicle conditions. Asterisk indicates significant difference (^∗^*p* < 0.05; ^∗∗^*p* < 0.01; and ^∗∗∗^*p* < 0.001, *t*-test). **(C)** AQP2 mRNA ratios summarized from the experiments shown in **(B)**. **(D)** Transepithelial resistance of the control and stable Elf3 knockdown cells grown on Transwell prior to the experiments. **(E,F)** Representative and summary of immunoblotting for AQP2 protein in the control and stable Elf3 knockdown cells in response to vehicle or dDAVP. Values are means ± SE, adjusted for loading (β-actin staining) and normalized against values in the control cells under the vehicle conditions. Cell lysate from hamster inner medullary collecting duct (IMCD) serves as a positive control. β-actin serves as a loading control.

To test whether Elf3 participates in vasopressin-induced AQP2 gene expression, Elf3 mRNA in the mpkCCD cells was knocked down with an shRNA sequence to about 0.33 ± 0.05, compared to the unity in the non-targeted control cells under the vehicle conditions (Figure 2A, shElf3 vs. shCon). In response to dDAVP, the Elf3 mRNA levels did not seem to change. In contrast, the AQP2 mRNA levels increased from the unity under the vehicle conditions to about 10.0 folds (9.99 ± 3.84) in the control cells upon dDAVP stimulation (Figure 2B, shCon). In the Elf3 knockdown cells (Figure 2B, shElf3), the basal AQP2 mRNA levels under the vehicle conditions were already lower (0.12 ± 0.04) than the unity in the control cells (shCon). In response to dDAVP, the AQP2 mRNA levels in the Elf3 knockdown cells increased to 1.11 ± 0.09, albeit at levels significantly lower than those in the control cells treated with dDAVP. However, the dDAVP-to-vehicle AQP2 mRNA ratios were not significantly different in the control and the Elf3 knockdown cells (Figure 2C). Thus, Elf3 does not mediate the vasopressin response but modulates basal as well as vasopressin-inducible levels of AQP2 mRNA expression. The above observations were not due to Elf3 knockdown that affected cell proliferation and polarization as the transepithelial resistance were similar in the control and the Elf3 knockdown cells (Figure 2D). Similar results were observed at the AQP2 protein level. As shown in Figure 2E, there was no detectable AQP2 protein in the control or Elf3 knockdown cells under the vehicle conditions. dDAVP induced AQP2 protein expression in the Elf3 knockdown cells although at a much lower level compared to that in the control cells. On average (Figure 2F), the AQP2 protein levels in the Elf3 knockdown cells (shElf3, 2.13 ± 0.11) were 2.2 folds lower than those in the control cells (4.67 ± 0.61) under the dDAVP conditions.

### Elf3 Overexpression Enhanced Basal and Vasopressin-Induced AQP2 Promoter Activity and mRNA Expression

**FIGURE 3:**
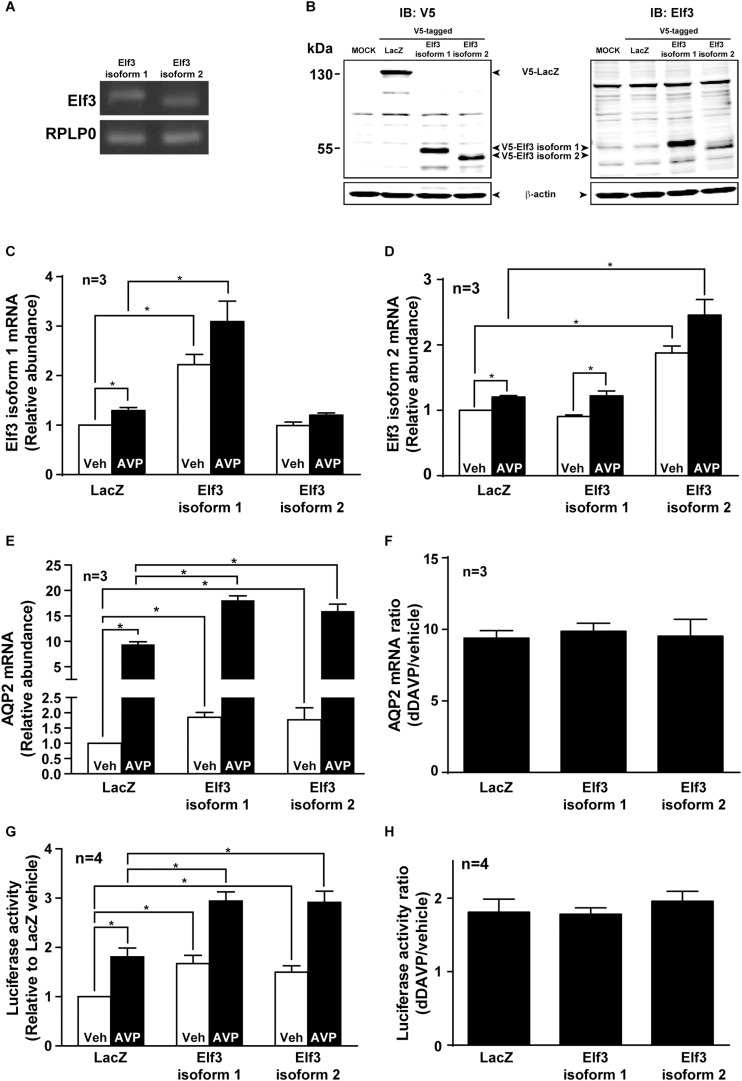
Elf3 overexpression enhanced basal and vasopressin-induced AQP2 promoter activity and transcription. **(A)** Expression of endogenous Elf3 isoform mRNA in the mpkCCD cells by RT-PCR and agarose gel electrophoresis analysis. The RPLP0 mRNA levels served loading control purposes. **(B)** Immunoblotting for Elf3 protein in the mpkCCD cells transfected with V5-tagged LacZ (β-galactosidase), V5-tagged Elf3 isoform 1, or V5-tagged Elf3 isoform 2 expression vector. β-actin staining served as a loading control. **(C)** Elf3 isoform 1. **(D)** Elf3 isoform 2. **(E)** AQP2 mRNA level in the mpkCCD cells transfected with LacZ, V5-tagged Elf3 isoform 1, or V5-tagged Elf3 isoform 2 expression vector. All cells were exposed to vehicle (Veh) or 0.1 nM dDAVP (AVP) for 8 h before mRNA measurements with quantitative RT-PCR. Values are means ± SE, adjusted for loading (RPLP0 mRNA levels) and standardized against the values in the control cells expressing LacZ under the vehicle conditions. Asterisk indicates significant difference (*p* < 0.05, *t*-test). **(F)** AQP2 mRNA ratios summarized from the experiments shown in **(E)**. **(G)** AQP2 promoter activity assay in the mpkCCD cells expressing LacZ, Elf3 isoform 1, or Elf3 isoform 2 after exposed to vehicle or dDAVP. **(H)** AQP2 promoter activity ratios summarized from experiments shown in **(G)**.

There are two Elf3 isoforms 1 and 2 expressed in the mpkCCD cells by RT-PCR analysis (Figure 3A). Elf3 isoform 2 is shorter than isoform 1 by 20 amino acids (54–73, Uniprot accession Q3UPW2) in the pointed domain that is not involved in DNA binding. To examine their functions in vasopressin-induced AQP2 gene expression, V5-tagged Elf3 isoform 1 or 2 was selectively overexpressed in the mpkCCD cells as detected by either a V5 antibody or Elf3 antibody (Figure 3B). Note that the background noise by the V5 antibody was lower than that of the Elf3 antibody and hence the V5 antibody was elected for subsequent experiments. Overexpression of the Elf3 isoform proteins was accompanized with elevated Elf3 isoform mRNA. Overexpression of Elf3 isoform 1 elevated the mRNA level of isoform 1, not isoform 2 (Figure 3C). Overexpression of Elf3 isoform 2 elevated the mRNA level of isoform 2, not isoform 1 (Figure 3D). In the cells overexpressing either Elf3 isoform (Figure 3E), the basal AQP2 mRNA levels i.e., under the vehicle conditions were already higher than that in the LacZ-expressing cells. In response to dDAVP, the AQP2 mRNA level increased to about 9.4 folds in the LacZ expressing cells (Figures 3E,F). To the same degree, the AQP2 mRNA levels in the Elf3 isoform expressing cells also increased about ninefolds (Figure 3F). Thus, Elf3 overexpression elevated basal and dDAVP-induced AQP2 mRNA levels without affecting the dDAVP-to-vehicle AQP2 mRNA ratio. The increases in the AQP2 mRNA levels reflected in the increases in the AQP2 promoter activity (Figure 3G). Overexpression of either Elf3 isoform already significantly elevated the basal AQP2 promoter activity under the vehicle conditions (Figure 3G, 1.67 ± 0.17 for Elf3 isoform 1 and 1.50 ± 0.13 for Elf3 isoform 2 expressing cells), compared to the unity in the LacZ-expressing cells. In response to dDAVP, the AQP2 promoter activity increased about twofolds in the cells overexpressing LacZ or either Elf3 isoform (Figure 3H). Thus, Elf3 overexpression elevated both basal and vasopressin-induced AQP2 promoter activity without affecting the dDAVP-to-vehicle AQP2 promoter activity ratios. Note that dDAVP induced small

increases in the Elf3 isoform mRNA in the LacZ-expressing cells (Figures 3C,D). This is probably due to LacZ overexpression and is irrelevant to vasopressin regulation as the endogenous Elf3 does not answer to vasopressin in the control cells (Figure 3A).

### Elf3 Did Not Translocate to the mpkCCD Cell Nuclei in Response to Vasopressin

**FIGURE 4:**
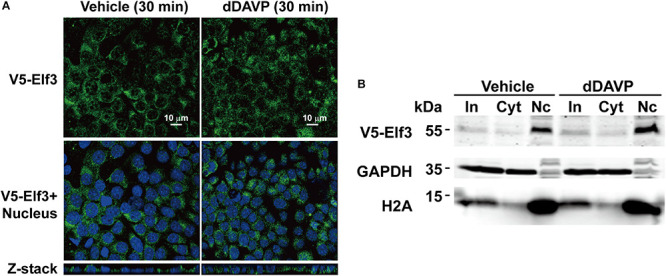
Vasopressin did not induce Elf3 nuclear translocation in the mpkCCD cells. **(A)** confocal immunofluorescence micrographs of mpkCCD cells transfected with the V5-tagged Elf3 isoform 1 expression vector and stimulated with dDAVP for 30 min. **(B)** Nucleus (Nc) vs. cytosol (Cyt) fractionation followed by immunoblotting for V5-tagged Elf3 in the mpkCCD cells under vehicle or dDAVP-stimulated conditions. GAPDH and histone H2A were stained as a cytosol and a nucleus marker, respectively. In, input i.e., cell lysate.

Because our proteomics results indicate an increased amount of Elf3 protein in the mpkCCD cell nuclei in response to vasopressin (Schenk et al., 2012), we examined intracellular localization of a V5-tagged Elf3 protein in the mpkCCD cells in response to dDAVP using confocal immunofluorescence microscopy. As shown in Figure 4A, the V5-tagged Elf3 isoform 1 was intracellular under the vehicle conditions and remained intracellular in response to dDAVP for 30 min. Nucleus vs. cytosol fractionation followed by immunoblotting showed similar results. The amount of V5-tagged Elf3 in the cytosol vs. nucleus did not change in response to dDAVP (Figure 4B). Note that the proteins of the nuclear fraction were 5 times enriched than those of the cytosolic fraction and hence the Elf3 signal was stronger in the nuclear fractin than in the cytosolic fraction. Our results are quite different from those reported by Mark Knepper’s group (Schenk et al., 2012). One apparent difference between the two experiments was the measurement of V5-tagged exogenous Elf3 in the present study vs. endogenous Elf3 in their study. We were not certain whether the V5 tag would interfere with nuclear translocation of V5-tagged exogenous Elf3. We were obliged to measure V5-tagged exogenous Elf3 with the V5 antibody because the antibody for endogenous Elf3 measurement had quite significant level of noises compared to those of the V5 tag antibody (Figure 3B).

### Elf3 Bound the Ets Element in the AQP2 Promoter

**FIGURE 5:**
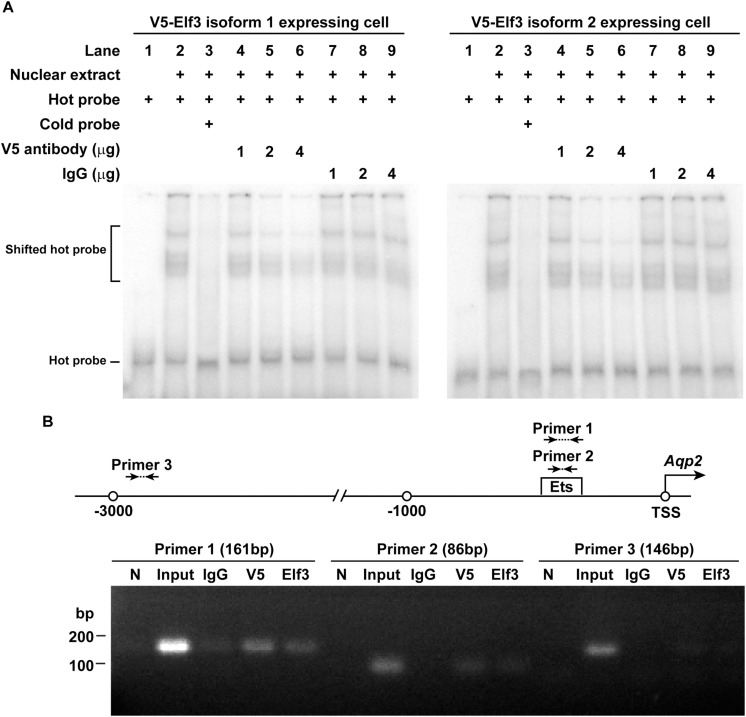
Elf3 bound the Ets element in the AQP2 promoter in the mpkCCD cells. **(A)** Electrophoretic mobility shift assay. Nuclear extract from either V5-tagged Elf3 isoform 1 (left panel) or V5-tagged Elf3 isoform 2 (right panel) expressing mpkCCD cells was incubated with a ^32^P isotope-labeled AQP2 promoter DNA (hot probe) and subjected to electrophoresis followed by autoradiography. A non-isotope labeled competitor AQP2 promoter DNA (cold probe) or a V5 antibody (at 1, 2, or 4 μg) was used to inhibit the interaction. A non-specific IgG was used as a control. **(B)** ChIP-PCR assay. Chromatin immunoprecipitation was done with a control (IgG), V5 tag, or Elf3 antibody before PCR analysis with primer sets framing the indicated regions in the AQP2 promoter. N is a no template negative PCR control.

Electrophoretic mobility shift assay was used to examine whether Elf3 binds AQP2 promoter containing an Ets element. Nuclear extract of the mpkCCD cells expressing either V5-tagged Elf3 isoform was incubated with a γ-^32^P isotope-labeled hot AQP2 promoter DNA before subjected to electrophoresis and autoradiography. The left panel in Figure 5A shows representative results using nuclear extract from the V5-tagged Elf3 isoform 1 expressing cells. The hot AQP2 promoter DNA was observed as a band in the lower portion of the gel (Lane 1). Incubating the hot DNA with the nuclear extract decreased the electrophoretic mobility of the DNA and resulted in a shift of the DNA to a higher position in the gel (Lane 2), indicative of a binding between the AQP2 promoter DNA and the V5-tagged Elf3. This mobility shift was inhibited when a non-isotope labeled cold AQP2 promoter DNA was included in the incubation to compete against the hot DNA for binding with the V5-tagged Elf3 (Lane 3). The mobility shift was reduced in a dose-dependent manner when a V5 antibody was included in the incubation to inhibit the interaction between the V5-tagged Elf3 and the hot AQP2 promoter DNA (Lanes 4–6). We believe that binding by the V5 antibody could alter the structure of the V5-tagged Elf3 protein and interferes with Elf3’s DNA binding ability as has been reported (Leers-Sucheta et al., 1997). Inclusion of a non-specific IgG in the incubation did not affect the mobility shift (Lanes 7–9). Similar results were observed in the mpkCCD cells expressing V5-tagged Elf3 isoform 2 (Figure 5A, right panel). Results from chromatin immunoprecipitation coupled with polymerase chain reaction support Elf3 binding to the AQP2 promoter in the vicinity of the Ets binding element. In the mpkCCD cells expressing V5-tagged Elf3 isoform 1 (Figure 5B), PCR products were amplified from the chromatin immunoprecipitated with either V5 tag or Elf3 antibody with two primer sets (1 and 2) surrounding the Ets binding element of the AQP2 promoter. The third primer set located at about 2,600 bp upstream to the Ets binding element did not produce any PCR product. Likewise, chromatin immunoprecipitated with an irrelevant IgG antibody also failed to produce PCR product.

### Ets Mutation Reduced Basal and Vasopressin-Induced AQP2 Promoter Activity

**FIGURE 6:**
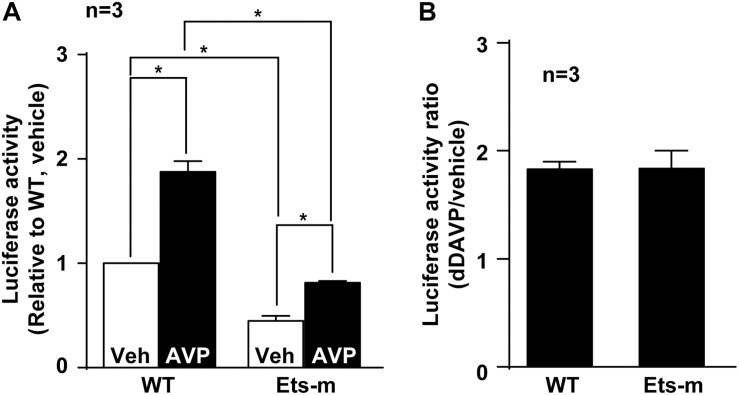
Ets mutation reduced basal and vasopressin-induced AQP2 promoter activity. **(A)** Wild type (WT) and Ets mutant (Ets-m) AQP2 promoter activity assay. WT or Ets-m AQP2 promoter (1,000 bp) was transfected into the mpkCCD cells before stimulated with vehicle (Veh) or dDAVP (AVP) prior to reporter activity assay. Values are means ± SE, normalized against values of the wild type promoter under the vehicle conditions. Asterisk indicates significance (*p* < 0.05, *t*-test). **(B)** Ratios of the AQP2 promoter activity in response to dDAVP vs. vehicle.

Because Elf3 binds the Ets element (Figure 5) and enhances basal and vasopressin-induced AQP2 promoter activity (Figure 3G), mutation in the Ets element that interferes with Elf3-Ets binding is expected to reduce both basal and vasopressin-induced AQP2 promoter activity. Indeed, the basal activity of the Ets mutant AQP2 promoter was significantly lower than that of the wild type promoter (Figure 6A). In response to dDAVP, the wild type AQP2 promoter activity increased to about 2 folds compared to that under the vehicle conditions (Figures 6A,B). Similarly, the Ets mutant AQP2 promoter also increased to about twofolds compared to that under the vehicle conditions (Figures 6A,B). These results are consistent with the idea that Elf3 modulates both basal and vasopressin-inducible AQP2 promoter activity thereby gauging vasopressin response.

### Lithium Chloride Reduced Elf3 and AQP2 mRNA in the mpkCCD Cells

**FIGURE 7:**
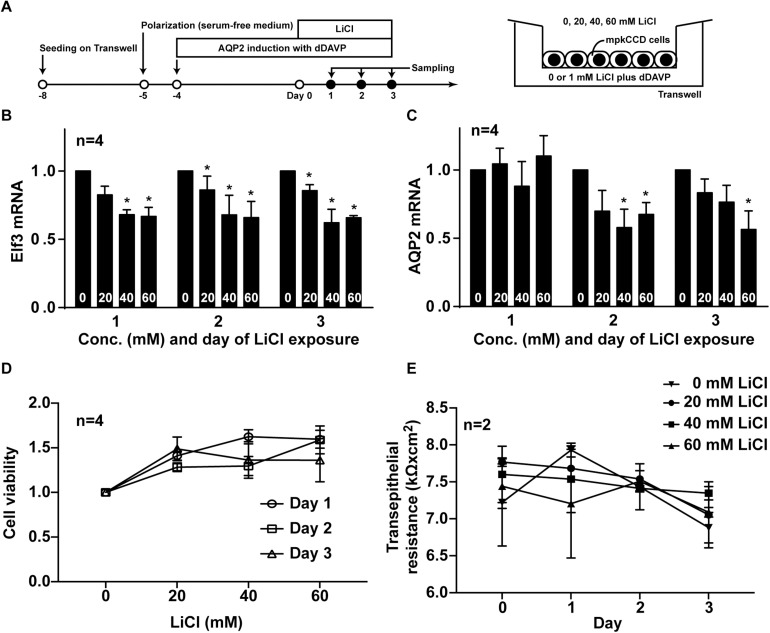
Lithium reduced Elf3 and AQP2 mRNA in the mpkCCD cells. **(A)** Experimental protocol. **(B)** Elf3. **(C)** AQP2 mRNA levels in the mpkCCD cells exposed to the indicated lithium chloride (LiCl) concentrations (0–60 mM) for 1–3 days. **(D)** Cell viability. **(E)** Transepithelial resistance values of the mpkCCD cells exposed to lithium chloride. Values are means ± SE, normalized against values of zero mM LiCl on the same day. Asterisk indicates significance (*p* < 0.05, *t*-test) against the values at zero mM LiCl on the same day. Two-way ANOVA was used to assess time- and dose-dependent effects of LiCl.

To test whether lithium-induced low AQP2 gene expression is a result of reduced Elf3 level, the mpkCCD cells were induced to express AQP2 before exposed to lithium chloride at varying concentrations for 3 days (Figure 7A). Both Elf3 (Figure 7B) and AQP2 (Figure 7C) mRNA levels were reduced in a time- and dose-dependent manner. Two-way ANOVA showed that lithium significantly reduced both Elf3 and AQP2 mRNA levels. Whereas there was not a significant time-dependent change in the Elf3 mRNA level by lithium, there was a significant time-dependent decrease in the AQP2 mRNA level by lithium. Note that the Elf3 mRNA level decreased on the first day after lithium treatment i.e., 1 day prior to AQP2 mRNA decrease. The Elf3 mRNA level did not further decrease with time upon lithium treatment. The AQP2 mRNA level decreased on the second day after lithium treatment and kept decreasing on the thrid day. These results are in line with a role of Elf3 in modulating AQP2 gene transcription. The above observations were associated with mild effects of lithium on cell viability (Figure 7D) and cell polarity (Figure 7E) based on two-way ANOVA.

### Lithium Chloride Reduced Elf3 and AQP2 mRNA in Mouse Kidney Inner Medulla

Encouraged by the *in vitro* cell line-based observations, we tested whether lithium chloride reduces Elf3 and AQP2 mRNA in mice fed with control vs. lithium-diet for 2 weeks. As summarized in Table 2, the mice on the lithium diet were normal in body weight, heart rate, mean blood pressure, and plasma osmolality as the control mice. However, the plasma lithium concentration was significantly elevated in the lithium-fed mice that produced 2.8 folds more urine with 2.2 folds lower osmolality than the control mice. The mice on the lithium-diet also consumed 1.6 folds more water than the control mice. The above effects were associated with reduced Elf3 (∼6 times less) and AQP2 (∼34 times less) mRNA levels in the lithium-fed mice, compared to controls.

**TABLE 2:** Physiological measurements in mice on control or lithium diet for 2 weeks.

	**Control diet (*n* = 3)**	**Lithium diet (*n* = 6)**
Body weight (g)	22.7 ± 0.5	21.6 ± 1.6
Heart rate (beat/min)	501.0 ± 67.5	494.4 ± 69.3
Mean blood pressure (mmHg)	86.4 ± 6.5	86.0 ± 8.0
Plasma Li^+^ concentration (mM)	<0.2	0.68 ± 0.1^∗^
Urine output (ml/day)	1.23 ± 0.32	3.42 ± 1.96^∗^
Water intake (ml/day)	2.9 ± 0.9	4.7 ± 0.7^∗^
Plasma osmolality (mOsm/L)	319.5 ± 3.1	319.5 ± 1.7
Urine osmolality (mOsm/L)	1281.2 ± 238.9	579.5 ± 149.0^∗^
**Critical cycle number**		
AQP2 mRNA	21.1 ± 1.2	26.2 ± 1.2^∗^
Elf3 mRNA	30.9 ± 0.9	33.5 ± 0.6^∗^

*^∗^Student’s *t* test against values from mice on the control diet, *p* < 0.05.*

## Discussion

Systems approaches including deep sequencing and protein mass spectrometry are very powerful modern tools for generating novel hypotheses concerning biological processes (Huling et al., 2012). Here, we followed up our prior systems findings (Yu et al., 2009; Schenk et al., 2012) with methods of molecular biology and biochemistry to test potential roles of the transcription factor Elf3 in vasopressin-induced AQP2 gene expression. In line with our hypothesis, we found that not only does Elf3 participate in AQP2 gene expression but it also modulates the responsiveness of the AQP2 promoter to vasopressin thereby gauging AQP2 gene expression. When the Elf3 amount is high as in the Elf3 overexpressing cells (Figures 3B–D), the basal AQP2 promoter activity i.e., under the vehicle conditions is elevated compared to that in the LacZ expressing cells (Figure 3G). Vasopressin induces a greater AQP2 promoter activity in the Elf3 overexpressing cells compared to that in LacZ overexpressing cells. However, the vasopressin-to-basal AQP2 promoter activity ratios remain constant (Figure 3H). The above Elf3 effects on the AQP2 promoter activity reflect in the AQP2 mRNA levels under both basal and vasopressin-stimulated conditions (Figures 3E,F). Again, the vasopressin-to-basal AQP2 mRNA ratios remain constant regardless Elf3 overexpression. Conversely, when the Elf3 amount is low as in the Elf3 knockdown cells (Figure 2A), the AQP2 mRNA levels under the basal and vasopressin-stimulated conditions are both lower than those in the control cells (Figure 2B). Yet, the vasopressin-to-basal AQP2 mRNA ratios remain constant regardless Elf3 knockdown (Figure 2C). Thus, Elf3 does not answer to vasopressin. It sets a basal tone of the AQP2 promoter activity that is amplified by vasopressin.

How could Elf3 set a basal tone for the AQP2 promoter? Elf3 apparently binds the Ets element in the AQP2 promoter (Figure 5). Hypothetically, Elf3 via its acidic protein domain could recruit the TATA box binding protein to facilitate transcription pre-initiation complex formation in the AQP2 promoter thereby promoting basal and vasopressin-stimulated AQP2 transcription (Chang et al., 1999). Alternatively, Elf3 may interact with the CBP/p300 protein complex that has histone acetyl transferase activity (Yang et al., 1998). This could result in remodeling of the chromatin structure locally at the AQP2 promoter in favor of basal AQP2 transcription under the vehicle conditions. The remodeled AQP2 promoter gains better accessibility to transcription factors that can be stimulated by vasopressin thereby enhancing AQP2 transcription (Radin et al., 2012). In line with this, mutation at the Elf3-binding Ets element reduces the AQP2 promoter activity under the basal as well as vasopressin-stimulated conditions (Figure 6A), again without affecting the vasopressin-to-vehicle response ratios (Figure 6B). In a recent systems study Isobe et al. (2017), predicted a similar regulatory model where vasopressin stimulates AQP2 transcription through induction of nuclear translocation of p300 acetyl transferase that increases histone H3K27 acetylation of vasopressin-responsive genes including AQP2. To the above model, our data add Elf3 that engages the CBP/p300 protein complex to modulate vasopressin responses with gauged AQP2 gene expression levels.

It is interesting to see that lithium chloride reduces Elf3 and AQP2 gene expression levels in the mpkCCD cells (Figure 7) and in the mouse inner medulla (Table 1). Lithium chloride is a frequently prescribed drug for bipolar diseases and other medical conditions (Vallon et al., 2019). One apparent side effect in the kidneys is lithium-induced diabetes insipidus characterized with polyuria and polydipsia associated with reduced AQP2 gene expression (Vallon et al., 2019). The underlying mechanisms have been extensively studied and involve multiple complex processes including reduced AQP2 transcription, interrupted vasopressin signaling, altered principal cell morphology, death, proliferation, and differentiation as well as vasopressin and cAMP independent processes (Moeller et al., 2013). The fact that lithium reduced cell viability (Figure 7D) and cell polarity (Figure 7E) might result from the about mechanisms. To these, our data provide another potential mechanism involving the transcription factor Elf3. One of the best-known targets of lithium is glycogen synthase kinase 3β (Rao, 2012). GSK3β inhibition activates transcription factor NF-κB that has a binding site in the Elf3 promoter region (Hoeflich et al., 2000; Rao et al., 2004). There are five NF-κB proteins of which three (RelA, NF-κB1 and NF-κB2) are highly expressed in the collecting duct cells (Uawithya et al., 2008; Yu et al., 2009; Hayden and Ghosh, 2012). NF-κB functions as homo- or heterodimer (Hayden and Ghosh, 2012). Depending on how they pair, the NF-κB dimers could either increase or decrease target gene expression (Hayden and Ghosh, 2012). Thus, lithium could potentially reduce Elf3 transcription via affecting NF-κB dimers. Reduced Elf3 transcription will reduce AQP2 gene transcription. This of course requires further evaluation.

In summary, the advent of modern systems biology approaches has benefited physiology studies with many novel and exciting hypotheses. In the present case, we successfully complemented our systems discovery with experimental evidence generated with biochemical and molecular biological approaches. By using conventional methods to test our own hypotheses generated in the systems studies, we discovered a new regulatory process unseen in our systems discoveries. In addition, our study also uncovered a potential new mechanism for lithium-induced diabetes insipidus via reducing the gene expression level of the transcription factor Elf3.

## Data Availability Statement

All datasets for this study are included in the manuscript/supplementary files.

## Author Contributions

M-JY conceived and coordinated the study, and wrote the manuscript. S-TL, C-CM, K-TK, W-LW, and T-HC were responsible for Figures 1–3. Y-FS and K-TK were responsible Figures 4, 7. S-CW was responsible for Figure 5A. S-HS was responsible for Figure 5B. S-TL and K-TK were responsible for Figure 6. C-HY and S-LL were responsible for Table 2. All authors reviewed the results and approved the final version of the manuscript.

## Conflict of Interest

The authors declare that the research was conducted in the absence of any commercial or financial relationships that could be construed as a potential conflict of interest.
